# Factors Associated with Hemorrhage of Melanoma Brain Metastases after Stereotactic Radiosurgery in the Era of Targeted/Immune Checkpoint Inhibitor Therapies

**DOI:** 10.3390/cancers14102391

**Published:** 2022-05-12

**Authors:** Eleni Zoga, Robert Wolff, Hanns Ackermann, Markus Meissner, Claus Rödel, Nikolaos Tselis, Georgios Chatzikonstantinou

**Affiliations:** 1 Department of Radiotherapy, Sana Hospital Offenbach, 63069 Offenbach am Main, Germany; eleni.zoga@sana.de; 2 Saphir Radiosurgery Center Frankfurt, 60528 Frankfurt am Main, Germany; robert.wolff@kgu.de; 3 Institute of Biostatistic and Mathematical Modeling, University Hospital, Goethe University Frankfurt, 60590 Frankfurt am Main, Germany; h.ackermann@add.uni-frankfurt.de; 4 Department of Dermatology, Venereology and Allergology, University Hospital, Goethe University Frankfurt, 60590 Frankfurt am Main, Germany; markus.meissner@kgu.de; 5 Department of Radiotherapy and Oncology, University Hospital, Goethe University Frankfurt, 60590 Frankfurt am Main, Germany; clausmichael.roedel@kgu.de (C.R.); nikolaos.tselis@kgu.de (N.T.)

**Keywords:** melanoma, brain metastases, hemorrhage, stereotactic radiosurgery, targeted therapy, immune checkpoint inhibitors

## Abstract

**Simple Summary:**

Melanoma brain metastases (MBM) have a high propensity for hemorrhage (HA) after treatment. Our retrospective analysis evaluated factors associated with HA of MBM after robotic stereotactic radiosurgery (SRS) in the era of modern systemic therapy, and to the best of our knowledge, this is the first study focusing on this side effect. A total of 55 patients with 279 MBM were treated. The use of anticoagulants was the only predictive factor, both for radiologically evident HA and HA causing grade 3 toxicity. The interval between the administration of systemic therapy and SRS was also significant with regard to HA causing grade 1 toxicity, but it appears that the combination was safe, at least concerning grade 3 toxicity. We believe that our study is a useful contribution to the current literature, as it provides insights regarding the factors that correlate with HA.

**Abstract:**

We aimed to evaluate the factors associated with hemorrhage (HA) of melanoma brain metastases (MBM) after Cyberknife stereotactic radiosurgery (SRS) in the modern era of systemic therapy. A total of 55 patients with 279 MBM were treated in 93 fractions. The median age, SRS dose, radiological follow-up, and time to HA were 60.4 years, 20 Gy, 17.7 months, and 10.7 months, respectively. Radiologically evident HA was documented in 47 (16.8%) metastases. Of the 55 patients, 25 (45.4%) suffered an HA. Among those, HA caused grade 3 toxicity in 10 patients (40%) and grade 1 symptoms in 5 patients (20%). Ten patients (40%) with HA experienced no toxicity. Logistic regression revealed the use of anticoagulants and the administration of systemic therapy within 7/15 days from SRS to be predictive for HA. When considering the HA causing grade 3 symptomatology, only the use of anticoagulants was significant, with the delivery of whole brain radiation therapy (WBRT) before the HA narrowly missing statistical significance. Our retrospective analysis showed that the administration of modern systemic therapy within 7/15 days from SRS may contribute to HA of MBM, though it appears safe, at least concerning grade 3 toxicity. The use of anticoagulants by the time of SRS significantly increased the risk of HA.

## 1. Introduction

Melanoma, with an average diagnosis age of 65 years [1], accounts for the third most common cause of brain metastases [2]. Among patients with metastatic disease, up to 40% present with or will develop brain metastases. The median survival for patients with untreated melanoma brain metastases (MBM) is only a few weeks [3]. Treatment for MBM includes surgical resection, whole-brain radiation therapy (WBRT), stereotactic radiosurgery (SRS), hypofractionated stereotactic radiotherapy (HFSR), systemic therapy, and a combination of these [4,5,6,7,8,9,10,11,12,13]. Nowadays, in patients with few asymptomatic metastases, initial treatment with SRS alone is preferred [14]. Traditionally, MBM have shown a poor response to chemotherapy due to low drug concentrations in the brain owing to the protective nature of the blood–brain barrier [15]. Novel targeted and immunotherapeutic agents have revolutionized the systemic management of melanoma. Current studies, using checkpoint inhibitors or targeted therapy agents, yielded intracerebral response rates as high as 58% [16,17].

Melanoma brain metastases have a strong propensity to bleed [18]. The rate of pre-treatment hemorrhagic MBM has been documented up to 35%, whereas the post-SRS hemorrhage (HA) rate has been noted up to 25% [19]. Hemorrhage can cause the acute onset of either a new focal neurological deficit or worsen pre-existing focal deficits [20]. Therefore, although for the majority of treated metastatic brain tumors the risk of spontaneous bleeding is acceptable and not further increased by careful therapeutic anticoagulation [21], in patients with MBM, therapeutic anticoagulation, in order to decrease venous thromboembolism-associated mortality/morbidity, which is particularly increased during the administration of immune checkpoint inhibitors (ICI) [22], should be balanced against the increased bleeding propensity.

Until now, most of the studies regarding the combination of SRS with immunotherapy or targeted-therapy agents have focused on the efficacy of the treatment [23]. Although the reported toxicity, especially high-grade toxicity, was low [24], little is known about parameters related to specific adverse events. In this report, we sought to investigate the factors associated with HA of MBM after SRS in the era of immunotherapy/targeted-therapy agents, particularly focusing on the possible impact of the interval between the delivery of SRS and the administration of systemic therapy.

## 2. Materials and Methods

Patients treated at our center with robotic SRS (Cyberknife^®^, Accuray, Sunnyvale, CA, USA) for MBM were included in this retrospective analysis. All patients had a histologically proven melanoma. Patients were included if they were diagnosed with either synchronous or metachronous MBM in a contrast-enhanced brain magnetic resonance imaging scan (MRI). Patients were excluded from the analysis if the MBM diagnosis was set solely according to a computed-tomography scan (CT) without the use of MRI, or if the follow-up brain imaging consisted only of CT scans. Patients with previous hemorrhagic MBM were also excluded, as these metastases intrinsically tend to rebleed after treatment [25]. Data regarding the administration of targeted/ICI therapies were retrieved from the Department of Dermatology of the university hospital. For patients receiving their targeted/ICI therapies in departments other than the Department of Dermatology of the university hospital, all treatment-related documents were collected. Written informed consent for the treatment was obtained from all patients. This retrospective analysis was approved by the local institute review board.

### 2.1. SRS Treatment Planning

A 1 mm thin slice planning CT of the brain was co-registered with a contrast-enhanced T1-weighted MRI of the same slice thickness and used for primary delineation of the gross target volume (GTV) and organs at risk. The planning target volume (PTV) was defined as the GTV without further margin; thus, PTV equaled GTV. Dedicated SRS thermoplastic masks were used for patient immobilization. Patient localization during treatment was carried out with stereoscopic X-ray image guidance. Treatment planning was performed using MultiPlan version 4.6.1 (Accuray, Sunnyvale, CA, USA). A mean GTV dose optimization was performed to generate Gamma Knife-like plans with high central tumor doses. Stereotactic radiosurgery was delivered in a single fraction.

### 2.2. Follow-Up and Statistical Analysis

Follow-up consisted of contrast-enhanced T1-weighted MRI scans every three months. Evidence of HA was based on non-contrast T1-weighted and susceptibility-weighted scans, along with T2 MRI sequences in addition to radiology reports. Hemorrhage was considered as such only if it was clearly stated so in the radiology report. Signs of hemosiderin without clear evidence of HA were not considered as HA. The primary outcome measure considered for the analysis was HA of MBM, particularly symptomatic HA necessitating hospitalization. If patients received more than one cycle of systemic therapy, the closest administration date to the SRS (either before or after SRS) was used for analysis. Toxicity was recorded according to the Common Terminology Criteria for Adverse Events version 5.0. A logistic regression model was used to evaluate the possible correlation between HA (radiologically evident and causing severe symptomatology > grade 2) and various parameters, such as SRS dose, volume of the treated metastases, HA with evident tumor progression or without progression, interval between the administration of systemic therapy and the day of SRS (either before or after SRS), use of anticoagulants, and the delivery of WBRT either before or after SRS but before the radiological evidence of the hemorrhagic MBM. Tumor response was assessed according to the RANO [26] and iRANO criteria [27]. A two-sided *p* value ≤ 0.05 was considered statistically significant. Statistical analysis was performed using BiAS software (Epsilon Verlag, Darmstadt, Germany).

## 3. Results

Between January 2015 and December 2019, 77 consecutive patients with a total of 320 MBM were treated with SRS. Three (3.8%) patients with cardiac pacemakers and no treatment planning MRI and two patients (2.5%) who were lost to follow-up after the treatment, as well as two (2.5%) patients who died within the first three months without having received any follow-up MRI, were excluded from the analysis. Additionally, 15 (19.4%) patients with previous hemorrhagic MBM or hemorrhagic MBM at diagnosis were also excluded, leaving 55 patients with a total of 279 MBM treated in 93 SRS fractions that were eligible for the analysis. The median age of the treated patients was 60.4 years (range, 29.9–84.9) with a median Karnofsky Performance Status of 90 (range, 60–100). Median radiological follow-up was 17.7 months (range, 1.6–84.8). Eight patients were receiving anticoagulants at the time of SRS, among which six (75%) were treated with acetylsalicylic acid, one (12.5%) with apixaban and another one (12.5%) with low-molecular-weight heparin. The tumor, SRS, and systemic therapy characteristics are summarized in Table 1, Table 2 and Table 3, respectively.

### 3.1. Patients and Administration of Systemic Therapy

Among all patients, 32 (58.2%), 14 (25.4%), 5 (9.1%), 3 (5.5%) and 1 (1.8%) received one, two, three, four and five SRS fractions for intracranial progression, respectively. Among the 279 irradiated lesions, 263 were treated with only one SRS fraction, irrespective of the total number of fractions every patient received for intracranial progression. Six patients received repeat treatment for 16 lesions overall, demonstrating infield recurrence. Among those 16 lesions, 9 lesions (56.2%) affecting one patient were re-irradiated before the occurrence of HA, whereas the remaining 7 (43.8%) in overall five patients were re-irradiated after the occurrence of HA.

Eight (14.5%) patients received WBRT, two (3.6%) before and six (6.4%) after SRS. Among the last six patients, WBRT was carried out before the diagnosis of SRS-treated hemorrhagic MBM in two patients, after a diagnosis in one of the patients, whereas the remaining three patients who also underwent WBRT after SRS encountered no HA from their SRS-treated MBM.

Concerning systemic therapy and its administration date with regard to SRS, ICI was administered 63 times, 54 times within 2 months before or after SRS and 9 times within >2 months before or after SRS. With reference to the SRS date, the median interval for the administration of ICI (either before or after SRS) was 10 days (range, 0–540). Immune checkpoint inhibitors were administered 34 times before SRS, 28 times after SRS and once on the SRS day. Targeted therapy was paused on the SRS day, as well as one day before and after in accordance with the Consensus Guidelines from the Eastern Cooperative Oncology Group [28].

### 3.2. Hemorrhage

The median time to radiologically evident hemorrhagic MBM was 10.7 months (range, 0.4–84.8). With reference to the overall MBM number, radiologically evident HA was documented in 47 out of the 279 (16.8%) metastases. In terms of patient cohort and irrespective of the metastases treated, 25 patients (45.4%) suffered a radiologically evident hemorrhagic metastasis. Among these, HA caused symptomatology, necessitating hospitalization (grade 3) in 10 patients (40%), while in 5 patients (20%), HA resulted in grade 1 symptoms. There were also 10 patients (40%) with radiologically evident hemorrhagic MBM suffering no symptomatology. Toxicity is summarized in Table 4. Of the 25 radiologically evident HA, 11 (44%) were accompanied by or attributed to local tumor progression, whereas the remainder 14 (56%) were evident under either stable disease or tumor regression. Concerning only the HA that caused toxicity grade 3, necessitating hospitalization, 4 out of 10 patients (40%) were accompanied by or attributed to local tumor progression, whereas in the remaining 6 patients (60%), it occurred under either stable disease or tumor regression.

### 3.3. Logistic Regression Model

Two logistic regression models with stepwise backward elimination were applied, one with HA as the dependent variable, irrespective of the grade of the symptomatology, and one with HA causing grade 3 symptomatology as the dependent variable. In the first case, and regarding the six factors evaluated, the use of anticoagulants by the time of SRS (*p* = 0.017) and the administration of the systemic therapy within seven days from the time of SRS (either before or after SRS) (*p* = 0.015) were proven to be statistically significant. When considering the HA-causing grade 3 symptomatology for analysis, the use of anticoagulants (*p* = 0.015) was the only parameter that was statistically significant, whereas the delivery of WBRT before the radiological evidence of hemorrhagic MBM did narrowly (*p* = 0.06) not reach statistical significance. Table 5 shows the two logistic regression models and the *p* value for the statistically significant factors.

## 4. Discussion

Our retrospective analysis evaluated the factors associated with HA of MBM after SRS in the era of modern systemic therapy. To the best of our knowledge, this is the first one focusing on this adverse event. In our study, we found that the use of anticoagulants was the only factor that significantly contributed to HA causing toxicity grade 3. When considering all the radiologically evident HA, irrespective of the grade of toxicity caused, the use of anticoagulants by the time of SRS and the administration of systemic therapy within 7 days from SRS (either before or after SRS) were significant factors. The same two factors were also statistically significant if, instead of 7 days, the systemic therapy administration was extended to 15 days, but not if the interval was further extended up to 30 days from SRS.

The use of anticoagulants was the only factor that significantly predicted HA causing high-grade toxicity, as well as HA irrespective of the toxicity grade caused. Among the eight patients receiving anticoagulants at the time of SRS, six (75%) presented with post-SRS HA, out of which five (83.3%) exhibited HA causing grade 3 toxicity. Of those five patients, four (80%) took acetylsalicylic acid. Contrary to our findings, the use of anticoagulants was not predictive of HA in the study of Lucas et al. [29], who treated a cohort comparable to ours, as well as in the study by Redmond et al. [30], although the last one did not include modern systemic therapy.

The other factor predictive of radiologically evident HA, irrespective of the grade of toxicity caused, was the administration of systemic therapy within 7 and 15 days from the time of SRS. Okoukoni et al. [31] came to the same conclusion, though without further specifying the grade of toxicity encountered, while Lucas et al. [29] did not notice any correlation. As this factor was no more statistically significant when considering grade 3 toxicity, it appears that modern systemic therapy for MBM can be safely administered with SRS, at least for HA causing high-grade toxicity.

In most of the studies concerning SRS and modern systemic therapy for MBM, HA was recorded in the context of overall toxicity, and no study to date has evaluated possible factors associated with it. For example, in the study by Ly et al. [32], who reported on the local control after SRS for 198 MBM with or without BRAF mutation in patients who were treated with a BRAF inhibitor and in those who were not, the 1-year rates of freedom from intratumoral HA were 39.3% and 77.0%, respectively (*p* = 0.0003). In a study by Ahmed et al. [33], which evaluated the clinical outcomes of 26 patients with 73 MBM predominantly treated with SRS and ICI, HA was noted in four lesions.

Currently, there have only been two studies [29,31] addressing the parameters impacting HA after SRS, partially including patients treated with modern systemic therapy, and, to date, they have only been published in abstract form. In the first study, by Lucas et al. [29], 128 patients with 428 MBM received SRS. In comparison with our study, patients who also presented with hemorrhagic MBM were included. Systemic therapy consisted of chemotherapy in 55% and biologic therapy in 45% of the patients, whereas 38% of the patients were anticoagulated. The median margin dose was 18.8 Gy. After a median follow-up of 6.5 years, HA occurred in 31% post-SRS. The only factor predictive for post-SRS HA was a decreasing margin dose. Patients with HA pre-SRS were 2.3 times more likely to have additional HA post-SRS (*p* = 0.06). In the second study, Okoukoni et al. [31] treated 107 patients with 548 MBM. The SRS dose was in median 20 Gy for a median PTV of 2.8 cm^3^. Seven and seventeen patients also received immunotherapy concurrently and within 1 year from SRS, respectively. After a median follow-up of 13.5 months, post-SRS HA was noted in 123 MBM (22%). Of the factors evaluated, PTV volume (*p* = 0.0001), total MBM volume (*p* = 0.0006), and the administration of immunotherapy (*p* = 0.04) were associated with an increased risk of HA. While the administration of immunotherapy also significantly impacted the risk of HA in our study, we did not find any correlation with the PTV treated, perhaps due to the smaller PTV that was irradiated (median 1.47 cm^3^).

There are some limitations in our study. Firstly, the retrospective nature with its intrinsic bias. Secondly, the relatively small sample size included patients receiving both targeted therapies and ICI, and thus precluded a further analysis of the correlation of each therapy with the risk of HA. Furthermore, we were not able to retrospectively record the dose of the anticoagulants, namely, therapeutic vs. prophylactic, which might have played an additional role in the rate of HA. Nevertheless, our study is the first to analyze the factors associated with HA of MBM in the modern era of systemic therapy. We particularly focused on the impact of the time between the administration of contemporary melanoma systemic therapy and the SRS by analyzing different intervals within one month. Moreover, we excluded patients with MBM who had encountered HA before SRS, an intrinsic factor for re-HA that could have possibly biased the true effect of the factors analyzed. As patient numbers grow and results mature, our aim is to perform and subsequently present an analysis separating targeted therapies from ICI in order to definitively elucidate the impact of each systemic treatment on HA of MBM.

## 5. Conclusions

Our study shows that HA of MBM may not that seldom lead to neurological toxicity, and should always be considered as a possible adverse event. Though the interval of the administration of modern systemic therapy and SRS may play a role regarding radiologically evident HA, it appears that its combination is safe, at least concerning HA causing high-grade toxicity. According to our findings, physicians should be alerted for the risk of HA after SRS and modern systemic therapy, especially in patients receiving anticoagulants.

## Figures and Tables

**Table 1:** Tumor characteristics.

Characteristic	N = 55 *n* (%)
Primary tumor known	41 (74.5)
Cancer of unknown primary	14 (25.5)
Specific mutations	
BRAF	22 (40)
NRAS	10 (18.1)
cKIT	1 (1.8)
Brain metastases	
Synchronous	14 (25.5)
Metachronous	41 (74.5)
Number of irradiated brain metastases pro SRS	
Median	2
Range	1–20
Volume of the irradiated brain metastases pro SRS	
Median	1.47
Range	0.08–7.8
Number of irradiated metastases receiving one SRS fraction	263
Number of irradiated metastases receiving two SRS fractions	16

Abbreviations: SRS: stereotactic radiosurgery.

**Table 2:** Stereotactic radiosurgery characteristics.

Characteristic	Median (Range)
Dose	20 (16–20)
Isodose	64 (59–75)
Dose max	29.9 (23.5–33.9)
Dose mean	24.1 (19.1–27.6)
Conformity index	1.15 (1.02–2.44)
Homogeneity Index	1.56 (1.17–1.69)
Coverage	95.5 (95.5–100)

**Table 3:** Characteristics of the systemic therapy administered timely closest to every SRS fraction.

Systemic Therapy	SRS Fractions *n* = 93 (%)
Immune checkpoint inhibitor	63 (67.7)
Targeted therapies	20 (21.5)
Dacarbazine	2 (2.2)
Interferon	2 (2.2)
No systemic therapy	6 (6.4)
Immune checkpoint inhibitor	
Nivolumab	19
Pembrolizumab	19
Ipilimumab	10
Ipilimumab/Nivolumab	15
Targeted therapy	
Dabrafenib/Trametinib	13
Vemurafenib/Cobimetinib	4
Dabrafenib	2
Vemurafenib	1
Administration of immune checkpoint inhibitor either before or after SRS	*n* = 63 (%)
Within 7 days	22 (34.9)
Within 15 days	37 (58.7)
Within 30 days	47 (74.6)

**Table 4:** Toxicity according to CTCAE v5.0.

Toxicity	N = 25 (%)
Grade 3	
Generalized seizure	4 (16)
Hemiparesis	3 (12)
Ataxia	2 (8)
Cognitive disturbance	1 (4)
Grade 1	
Headache	4 (16)
Facial nerve disorder	1 (4)
Grade 0	10 (40)

Abbreviations: CTCAE: Common Terminology Criteria for Adverse Events.

**Table 5:** Logistic Regression model with dependent variable. (**a**) radiological evident hemorrhage. (**b**) hemorrhage causing toxicity ≥ grade 3.

a			
Independent Variable	Standard Deviation	*p* Value	Confidence Interval
Systemic Therapy within 7 days from SRS	0.46	0.015	1.34—16.3
Receipt of Anticoagulants by the day of SRS	0.63	0.017	1.63—161.7
**b**			
**Independent Variable**	**Standard Deviation**	***p*** **Value**	**Confidence Interval**
Receipt of Anticoagulants by the day of SRS	0.95	0.01	1.77—75.2
WBRT before the evidence of hemorrhage	1.39	0.06	0.89—209.2

Abbreviations: SRS: Stereotactic Radiosurgery, WBRT: Whole Brain Radiotherapy.

## Data Availability

Patient data will be provided by the corresponding author upon request.
